# Folate Receptor-α (FOLR1) Expression and Function in Triple Negative Tumors

**DOI:** 10.1371/journal.pone.0122209

**Published:** 2015-03-27

**Authors:** Brian M. Necela, Jennifer A. Crozier, Cathy A. Andorfer, Laura Lewis-Tuffin, Jennifer M. Kachergus, Xochiquetzal J. Geiger, Krishna R. Kalari, Daniel J. Serie, Zhifu Sun, Alvaro Moreno Aspita, Daniel J. O’Shannessy, Julia D. Maltzman, Ann E. McCullough, Barbara A. Pockaj, Heather E. Cunliffe, Karla V. Ballman, E. Aubrey Thompson, Edith A. Perez

**Affiliations:** 1 Department of Cancer Biology, Mayo Clinic, Jacksonville, Florida, United Sates of America; 2 Department of Hematology and Oncology, Mayo Clinic, Jacksonville, Florida, United States of America; 3 Department of Pathology and Laboratory Medicine, Mayo Clinic, Jacksonville, Florida, United States of America; 4 Department of Health Sciences Research, Mayo Clinic, Rochester, Minnesota, United States of America; 5 Department of Health Sciences Research, Mayo Clinic, Jacksonville, Florida United States of America; 6 Department of Biomedical Statistics and Informatics, Mayo Clinic, Rochester, Minnesota, United States of America; 7 Department of Translational Medicine and Diagnostics, Morphotek, Exton, Pennsylvania, United States of America; 8 Department of Clinical Development, Morphotek, Exton, Pennsylvania, United States of America; 9 Department of Laboratory Medicine and Pathology, Mayo Clinic, Scottsdale, Arizona, United States of America; 10 Department of Laboratory Medicine and Pathology, Mayo Clinic, Scottsdale, Arizona, United States of America; 11 Department of Pathology, Dunedin School of Medicine, University of Otago, Dunedin, New Zealand

## Abstract

Folate receptor alpha (FOLR1) has been identified as a potential prognostic and therapeutic target in a number of cancers. A correlation has been shown between intense overexpression of FOLR1 in breast tumors and poor prognosis, yet there is limited examination of the distribution of FOLR1 across clinically relevant breast cancer subtypes. To explore this further, we used RNA-seq data from multiple patient cohorts to analyze the distribution of FOLR1 mRNA across breast cancer subtypes comprised of estrogen receptor positive (ER+), human epidermal growth factor receptor positive (HER2+), and triple negative (TNBC) tumors. FOLR1 expression varied within breast tumor subtypes; triple negative/basal tumors were significantly associated with increased expression of FOLR1 mRNA, compared to ER+ and HER2+ tumors. However, subsets of high level FOLR1 expressing tumors were observed in all clinical subtypes. These observations were supported by immunohistochemical analysis of tissue microarrays, with the largest number of 3+ positive tumors and highest H-scores of any subtype represented by triple negatives, and lowest by ER+ tumors. FOLR1 expression did not correlate to common clinicopathological parameters such as tumor stage and nodal status. To delineate the importance of FOLR1 overexpression in triple negative cancers, RNA-interference was used to deplete FOLR1 in overexpressing triple negative cell breast lines. Loss of FOLR1 resulted in growth inhibition, whereas FOLR1 overexpression promoted folate uptake and growth advantage in low folate conditions. Taken together, our data suggests patients with triple negative cancers expressing high FOLR1 expression represent an important population of patients that may benefit from targeted anti-FOLR1 therapy. This may prove particularly helpful for a large number of patients who would typically be classified as triple negative and who to this point have been left without any targeted treatment options.

## Introduction

Every year, worldwide, more than a million breast cancer cases are diagnosed [1]. Deaths have been declining due to better early detection strategies and improved therapies, particularly those targeted to specific biomarkers. Despite these improvements, there are approximately 230,000 deaths per year worldwide due to breast cancer, including about 40,000 deaths in the United States [1].

Breast cancer is currently grouped into three main clinically relevant molecular subtypes: hormone receptor positive [estrogen receptor (ER+) and/or progesterone receptor (PR+)], human epidermal growth factor receptor positive (HER2+), and triple negative (TNBC) which is ER-, PR-, and HER2- [2]. ER+ tumors account for nearly 70% of invasive breast cancers diagnosed, and the widespread use of ER antagonists in this subset of patients account for the bulk of the treatment-related decreases in mortality. The remaining 30% of breast cancers are closely split between HER2+ breast cancers and triple negative breast cancers, both of which have a worse prognosis compared to ER+ disease. Use of trastuzumab (a humanized monoclonal antibody against HER2) has improved survival in women with HER2+ breast cancer [3–5]. There is currently no known specific targeted therapy for triple negative breast cancers that significantly affects survival [6] although there is emerging evidence that this subgroup of breast cancers is heterogeneous [7–9].

A biomarker of recent interest in the cancer field is folate receptor alpha (FOLR1), a membrane-bound protein with high affinity for binding and transporting folate into cells. Folate is a necessary component of cell metabolism. Overexpression of FOLR1 may confer a growth advantage to tumors by increasing folate uptake and/or may affect cell proliferation via alternative cell signaling pathways [10–12]. FOLR1 levels have been found to be elevated in tumors of epithelial origin compared to normal tissue, including ovarian, breast, brain, lung and colorectal cancers [13–17]. The tumor specificity of FOLR1 makes it a promising target for diagnosis and treatment strategies. Several types of folate receptor targeted therapies, such as antibodies and folic acid-drug conjugates, have been developed and are in various phases of clinical trials for treatment of ovarian and lung cancer [18]. Farletuzumab (MORAb-003) is a monoclonal anti-FOLR1 antibody that elicits antibody dependent cellular cytotoxicity (ADCC) [19]. Vintafolide (EC145) is a folate-conjugated small molecule designed to deliver the chemotherapeutic drug vinblastine selectively to cells expressing the folate receptor [20]. Moreover, diagnostic FR-targeted imaging agents have become available to help select patients with FR-expressing tumors likely to respond to folate receptor-targeted therapies [21, 22].

To date, much of the focus on folate receptor in cancer has been directed towards ovarian cancer. However, several studies in breast cancer suggest that FOLR1 may also represent an important therapeutic target in primary breast cancers. In 2007, Hartmann and coworkers first reported a strong correlation between intense overexpression of FOLR1and early recurrence of breast cancer [23]. Since then, two laboratories have provided evidence indicating FOLR1 expression is significantly associated with triple negative and ER negative subtypes [24, 25]. However, the significance of these findings has been limited by investigation into a relatively small set of clinically-relevant breast cancer subtypes and use of only immunohistochemistry for measurement. Moreover, discrepancies have emerged on the association of FOLR1 with clinicopathological parameters, likely arising from inter-laboratory differences in scoring criteria. Independent validation of these studies with larger clinical populations and by additional technical means of measuring FOLR1 expression will provide useful information into understanding the extent of FOLR1 overexpression in breast cancer subtypes and its association with clinicopathological parameters. Moreover, additional studies may provide insight into whether FOLR1 expression is associated with a particular subtype of triple negative cancer and what the biological consequences of FOLR1 overexpression are in breast cancer?

To confirm and expand the understanding of the role of FOLR1 in breast cancer, we compared expression of FOLR1 at the RNA and protein level in different subtypes of human breast cancer [(hormone receptor positive (ER+/PR+), HER2+, and TNBC] using both next generation sequencing and immunohistochemistry methods. We demonstrate that both FOLR1 mRNA and protein abundance varies widely among subtypes of breast cancer, with subpopulations of each subtype expressing high FOLR1. Importantly, triple negative tumors were significantly enriched in FOLR1 abundance compared to ER+ and HER2+ tumors. However, no specific subtype of triple negative tumors (i.e. BL1, BL2, IM, M, MS, LAR [9]) was significantly enriched for FOLR1 expression. We further show that knockdown of FOLR1 inhibits growth of triple negative cell lines, and its overexpression promotes folate uptake and a growth advantage in a low folate environment. Taken together, our data suggest that patients with triple negative cancers expressing high FOLR1 expression represent an important population of patients to consider for clinical trials evaluating targeted anti-FOLR1 therapy.

## Materials and Methods

### Tissue microarrays (TMAs)

Tissue microarrays (TMAs) were constructed from 131 breast cancer tumors selected from women with a range of pathological breast diagnoses, including ductal carcinoma in situ (DCIS, n = 4), estrogen receptor positive (ER+, n = 33), human epidermal growth factor receptor positive (HER2+, n = 26), and triple negative breast cancer (TNBC, n = 68). TMAs were constructed from formalin fixed paraffin embedded (FFPE) tissue blocks in the Mayo Clinic Tumor Registry, from tumor samples reviewed by our study pathologists (XJG, AEM). Arrays were constructed from suitable small (0.6 mm in diameter) punches using a manually operated tissue microarrayer (ATA-100 Advanced Tissue Arrayer, Millipore). Multiple cores from each patient’s tumor block were transferred into the recipient block. Additionally cores of serous ovarian cancer and liver were added to act as controls for immunohistochemistry markers. All analytical procedures were reviewed and approved by the Mayo Clinic Institutional Review Board.

### Immunohistochemisty

Immunohistochemistry (IHC) was performed on FFPE tissue microarrays using a MACH4 Universal HRP-Polymer Detection Kit (Biocare Medical) as previously described [26]. FFPE TMA specimens were sectioned at 5 microns, placed on positively-charged glass slides and heated at 60°C for at least one hour. Slides were deparaffinized in sequential baths of xylene, transferred to sequential baths of 100% ethanol, followed by sequential baths of 95% ethanol and then rinsed in deionized (DI) water. The IHC procedure involves pretreatment of slides in Diva heat-induced epitope retrieval solution (Biocare Medical) inside a pressurized decloaking chamber with DI water and a pressurized incubation period at elevated temperature (125°C at 16 psi for 30 sec) followed by a 15 min of cooling to 95°C. The slides were then cooled at room temperature, washed in sequential baths of Tris buffered Saline/0.1% Tween-20 wash buffer (TBST). Slides were blocked using Peroxidase-1 blocking solution (Biocare Medical), washed with TBST buffer and blocked with a serum-free universal blocking reagent. Slides were incubated with the primary FOLR1 antibody mAb 26B3.F2 (Morphotek) at 1:500 dilution in antibody diluent (Dako) or with Bond Negative Mouse ready-to-use negative control antibody (Dako, for negative isotype tissue) for 60 min at room temperature. Slides were washed with TBST buffer and then incubated with MACH4 Mouse Probe Primary Antibody Enhancer (Biocare Medical) for 15 min, and then Universal Polymer-HRP reagent (Biocare Medical) for 20 min. After additional TBST washes, slides were incubated with a 3,3’-diaminobenzidine tetrahydrochloride (DAB) solution (Dako), rinsed and counterstained with hematoxylin. Slides were washed with water, dehydrated with sequential baths each of 95% and 100% ethanol and then sequential baths of xylene before coverslips were applied.

### IHC scoring

Digital images of the stained TMA slides were obtained using an Aperio ScanScope Image Scanner (Aperio Technologies). TMAs were evaluated using a semi-quantitative scoring method. A pathologist (XJG) scored membrane staining as negative (0), weak (1+), moderate (2+) and strong (3+) membrane staining; blinded to pathological tumor subtype. The percent of cells within each tissue core stained at each intensity was recorded to calculate an H-score for each sample. The H-score is a weighted score that captures both the proportion of positive staining and its intensity, and thus more representative of the staining of the entire tumor section. The H-score for staining each sample was defined as: *Hscore* = 3 * (% *at* 3 +) + 2 * (% *at* 2 +) + 1 * (% *at* 1 +) + 0 * (% *at* 0). H-score values can range from zero (no membrane staining) to a maximum of 300 (100% membrane staining at 3+). H-scores for each patient sample were averaged over 3 TMA cores. Comparison of H-scores among breast cancer subtypes was achieved using the Mann-Whitney test.

### Patient samples

Archival fresh frozen breast tumor tissue from patients diagnosed with breast cancer was obtained from the Mayo Clinic Tumor Registry. All tumors were obtained from surgical resection and were macrodissected to remove normal tissue prior to freezing. Pathologic diagnosis and molecular classification of breast carcinoma was diagnosed by board certified pathologists (XJG, AEM). Receptor status was identified by traditional immunohistochemical staining for ER, PR, and HER-2 protein. In some cases, HER2 protein status was equivocal, HER2 status was confirmed by amplification of the *HER2* gene by fluorescence in situ hybridization.

### Ethics statement

All breast tumor samples were collected according to a protocol that was approved by the Mayo Clinic Institutional Review Board with written informed consent, and were de-identified for this work. All samples were used in compliance with the patient-approved institutional IRB approved study protocols.

### Next generation sequencing

Breast cancer samples were obtained from the Mayo Clinic Tumor Registry as 5 μM frozen slices. RNA was extracted from 10 slices of each sample with the RNAeasy isolation kit (Qiagen). Libraries for the 31 triple negative cancer set were prepared with the Nugen Ovation RNA-Seq system. Sequencing was carried out at Mayo Clinic Advanced Genomic Technology Center at Rochester, MN, USA using the Illumina Genome Analyzer II (GA II). The Illumina standard pipeline, GA II was employed for processing of raw images, to make base calls and to generate FASTQ sequence reads from paired-end RNA-sequencing data. The exon–exon boundary database was generated using exon and gene definitions obtained from UCSC refFlat table for hg19 assembly. Uni-directional combinations of exon junction database for the sequencing length (50 bases) were generated using exon boundaries defined by the refFlat file from UCSC Table Browser. FASTQ sequence reads were aligned to the human reference genome (hg19) and to our in-house exon junction database using BWA. BWA is a fast and accurate short read aligner. A maximum of two mismatches were allowed for the first 32 bases in each alignment, and reads that had more than two mismatches or were mapped to multiple genomic locations (alignment score less than 3) were discarded. The aligned sequence tags were summarized and annotated using SnowShoes, an in-house RNA-Seq pipeline [27]. The read counts for genes are generated for further downstream analyses. Individual read count data were normalized using mode, as follows: read count/sample read count mode *sm, where sm is the smallest mode across all the samples [28].

### The Cancer Genome Atlas (TCGA) RNA-seq data

RNA-sequencing data for 843 invasive breast carcinomas was obtained from the cancer genomics hub data repository (https://cghub.ucsc.edu/). Alignment of RNA-Seq reads for samples was performed using Bowtie. Bowtie is a fast memory efficient, short sequence aligner. It aligns reads to the genome and maps them to a genome assembly [29]. Unaligned reads from Bowtie are used by TopHat to map them to splice junctions [30]. After initial quality control and alignment, we obtained binary alignment mapping (bam) files for 841 breast cancer samples. The breast cancer classification subtypes for all samples was obtained from the TCGA group. The TCGA group used PAM50 subtype classifier method to call the breast cancer subtypes. The 841 samples were classified into basal (n = 115), HER2+ (n = 59), Luminal A (n = 351), Luminal B (n = 166) subtypes, normal (n = 92), and unclassified (n = 58). Gene abundance data for RNA-Seq samples was estimated using HTSeq software (http://www-huber.embl.de/users/anders/HTSeq/doc/overview.html). HTSeq performs quality assessments and reports read-level counts for genes. Gene count data (n = 23,114 genes) were mode normalized and log2 transformed.

### RNA-seq data—Shah et al. [31]

RNA-sequencing data for 80 classified triple negative invasive breast carcinomas were obtained from Shah and coworkers [31]. The aligned bam files (hg18) were converted to fastq and then analyzed using our mRNA-seq analytical pipeline. Briefly, fastq files were aligned to the human genome hg19 using TopHat v1.4 with Bowtie v0.12.7 [30]. HTSeq v0.5.3p3 (http://www-huber.embl.de/users/anders/HTSeq/doc/overview.html) was used to perform gene level expression counting while BEDTools v2.7.1 [32] was used to count the reads mapping to individual exons. The gene level expression was normalized by per million of mapped reads (RPM) to annotated RefSeq genes. This normalized data were log2 transformed for comparison and statistical analysis.

### GEO expression analysis

A cohort of 129 primary breast cancer gene expression profiles generated using the Affymetrix U133plus2 platform was downloaded from Gene Expression Omnibus (GEO accession number: GSE5460) [33]. The data were log2 transformed and differential expression of FOLR1 examined between the two classes (ER+, n = 76, ER-, n = 53) using the two sided unpaired t-test, assuming unequal variances.

### Cell lines

All breast cancer cell lines were purchased from ATCC and maintained according to the manufacturer’s recommended instructions. Knockdown cell lines were created by transducing the desired cell line with recombinant lentivirus encoding nontemplate (NT) or FOLR1 shRNA (Sigma-Aldrich). Populations of stably transfected cells were selected in 5 μg/ml puromycin for three days prior to experimentation. The HS578T-FOLR1 cell line was created by infection with the retrovirus pLNCX-FOLR1, which encodes the full length human FOLR1 isoform #4. Cells were selected as a population and maintained in 1000 μg/ml of G418 for one week prior to use in experiments.

### Growth analysis of cell lines

For the MTT assay, cells were plated at 5,000 cells/well in quadruplicate onto 96 well clear bottom microplates. At each time point, cells were incubated with 1 mg/ml MTT (Sigma-Aldrich) at 37°C, 5% CO_2_ for 1 hour. Formazan precipitate (produced in proportion to the number of live cells) was then extracted from the cells by replacing the media on the cells with 200 μl/well DMSO (Sigma-Aldrich). The absorbance at 550 nm was then determined for each sample. For growth analysis via the BrdU incorporation assay, cells were in plated at 7,500 cells/well in quadruplicate onto black 96 well assay plates. After 1 hr to allow adherence, cells were treated with 10 μM BrdU for 24 hr. BrdU incorporation was measured by fluorescent elisa using the HTS BrdU Cell Proliferation Assay (BD Biosciences) as instructed. All data were plotted as mean +/− standard deviation. For measurement of apoptosis, cells were in plated at 7,500 cells/well in quadruplicate onto black 96 well assay plates and cultured for 48 hrs. Apoptosis was measured using the Caspase-Glo 3/7 luminescent assay according to the manufacturer’s instructions (Promega).

### Quantitative RT-PCR

Two-step quantitative reverse transcriptase-mediated real-time PCR (qPCR) was used to measure the abundance of individual mRNAs. RNA was extracted from cells with the RNAqueous kit as detailed by the manufacturer (Ambion). Equal aliquots of total RNA from samples were converted to cDNA with the High-Capacity cDNA Archive kit (Applied Biosystems), and qPCR reactions were performed in triplicate with 10 ng of cDNA and the TaqMan Universal PCR master mix (Applied Biosystems). All primer/probe sets were purchased from Applied Biosystems. All amplification data were collected with an Applied Biosystems Prism 7900 sequence detector and analyzed with Sequence Detection System software (Applied Biosystems). Data were normalized to the endogenous control POLR2A [34], and mRNA abundance was calculated using the ΔΔCT method [33].

### Folate uptake assay

Cell cultures in logarithimic growth phase were first washed three times in PBS, acid stripped in 0.15M NaCl, pH 3.5 for 2 min, and washed again 3X with PBS. Cells were cultured for 24 hrs in folate free RMPI media supplemented with 10% dialyzed FBS. Cell media was then replaced with folate free RMPI media supplemented +/- 1 uM FolateRSense (Perkin Elmer). After 1 hr of exposure to FolateRSense, cells were washed 3X with PBS, acid stripped 2X at 2 min, and washed again 3X with PBS. Cells were resuspended in PBS and analyzed via flow cytometry using a BD FACS Aria SORP, using the 640 laser for FolateRSense excitation and detection in the APC channel (670/30 BP filter). The main population of cells was gated on FSC-A vs. SSC-A, displayed as FSC-A vs. APC-A (FolateRSense). Cells were further gated on the FolateRSense positive Hs758T population.

## Results

### RNA-seq analysis identifies triple negative tumors with high FOLR1 expression

**Fig 1:**
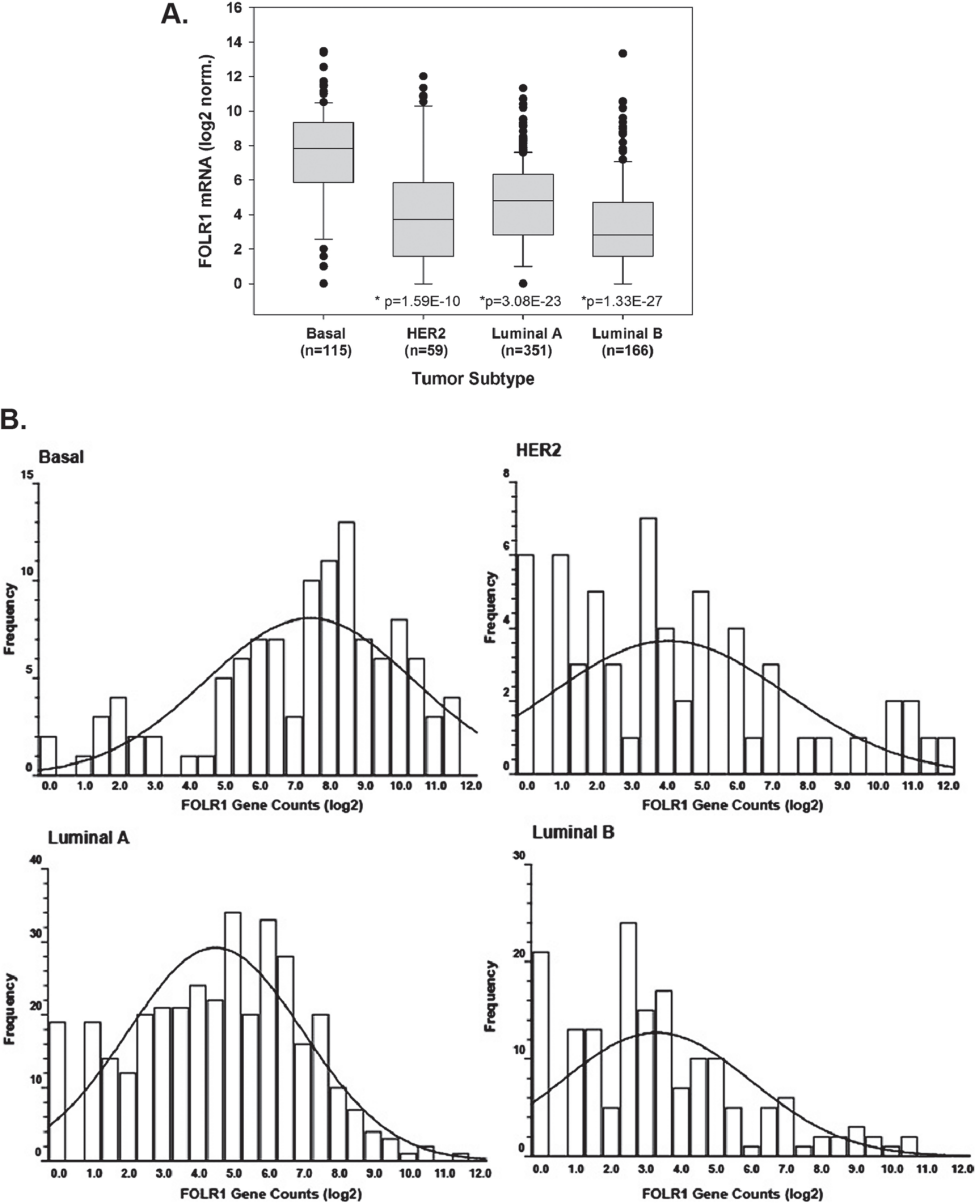
RNA-seq analysis of FOLR1 expression across clinically relevant breast cancer subtypes. (A). Log2 transformed data of FOLR1 abundance from a TCGA dataset of 691 breast cancers classified as basal, Luminal A, Luminal B, and HER2+. Bars represent 95% confidence levels for difference between the means. *Statistical significance calculated by two sided unpaired t-test, assuming unequal variances of basal versus Luminal A, Luminal B, and HER2+. (B). Distribution of FOLR1 expression across tumors of each subtype. Binned data shows frequency of tumors relative to log2 FOLR1 gene counts. Right shift in the trendline of the basal subtype illustrates a higher proportion of tumors have elevated FOLR1 levels.

**Fig 2:**
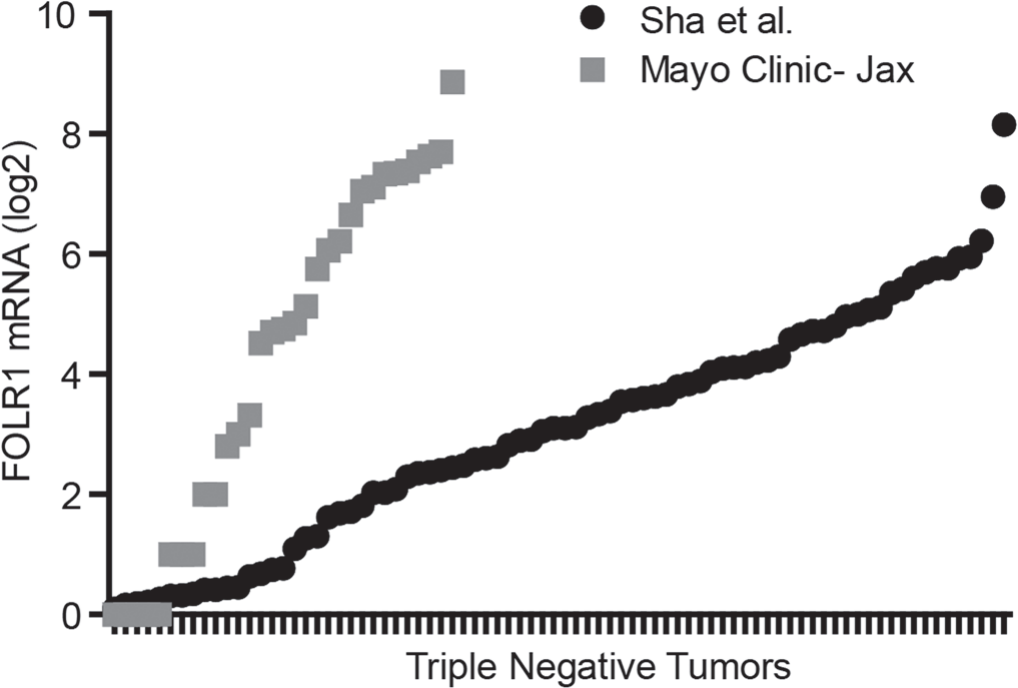
RNA-seq analysis of independent TNBC tumor datasets. Distribution of FOLR1 gene counts (log2) from RNA-seq analysis of 31 TNBC tumors (Mayo Clinic) and 80 triple negative tumors from RNA-seq dataset of Shah et al. [31]. Of note, gene counts cannot be compared across the independent tumor datasets as data were generated differently (library protocols, sequencing depth, normalization etc.).

We examined the distribution of FOLR1 mRNA using a TCGA RNA-seq dataset from 691 breast tumors classified into basal, Luminal A, Luminal B, and HER2+. Fig. 1A, B illustrate that FOLR1 levels in basal tumors were significantly higher than the other three subtypes. The data in Fig. 1A and 1B are also summarized in “violin” plots in S1 Fig. The expression of FOLR1 in the basal subtype is largely represented by the triple negative subtype which comprises 85% of the basal tumors (S2 Fig.). Fig. 1A and 1B also illustrates that subpopulations of each subtype express high FOLR1 mRNA abundance. In this context, “high” FOLR1 is defined as FOLR1 mRNA at or above the upper quartile limit of the basal population, or log2>9.3 for this dataset. The frequency of these subpopulations among subtypes can be observed in the distribution plot of Fig. 1B. It is clear that the higher median expression of FOLR1 in basal/triple negative population is reflected, in part, by a greater number of these high FOLR1 expressing tumors, despite a relatively wide range of expression (Fig. 1A and 1B). To further investigate the range of FOLR1 expression in triple negative breast cancers, we analyzed mRNA-seq expression data for FOLR1 on two additional TNBC cohorts, one from Mayo Clinic-Jacksonville (n = 31) and the other published by Shah et al. (n = 80, [31]). Consistent with the data above, expression of FOLR1 was widely distributed across triple negative tumors in both datasets (Fig. 2), with subpopulations of the tumors expressing high (i.e. >upper quartile limit) FOLR1 mRNA.

FOLR1 levels also appeared to correlate with estrogen receptor status. In TCGA samples, the ER- subset of basal samples (89% of all basal samples) expressed significantly higher FOLR1 than the ER+ subset (97%) of Luminal A and Luminal B tumors (S3 Fig., *P*<0.0001). This association was validated in a cohort of 128 primary breast cancers (ER+, n = 76; ER-, n = 53) gene expression profiles downloaded from Gene Expression Omnibus (GEO accession number: GSE5460) [33]. Comparison of the log2 fold change of the mRNA between ER+ vs. ER− sample groups demonstrated significantly higher median expression in ER- tumors (*P* = 0.013, S4 Fig.).

### Immunohistochemical analysis of tissue microarrays confirms triple negative tumors are enriched for FOLR1 expression

**Fig 3:**
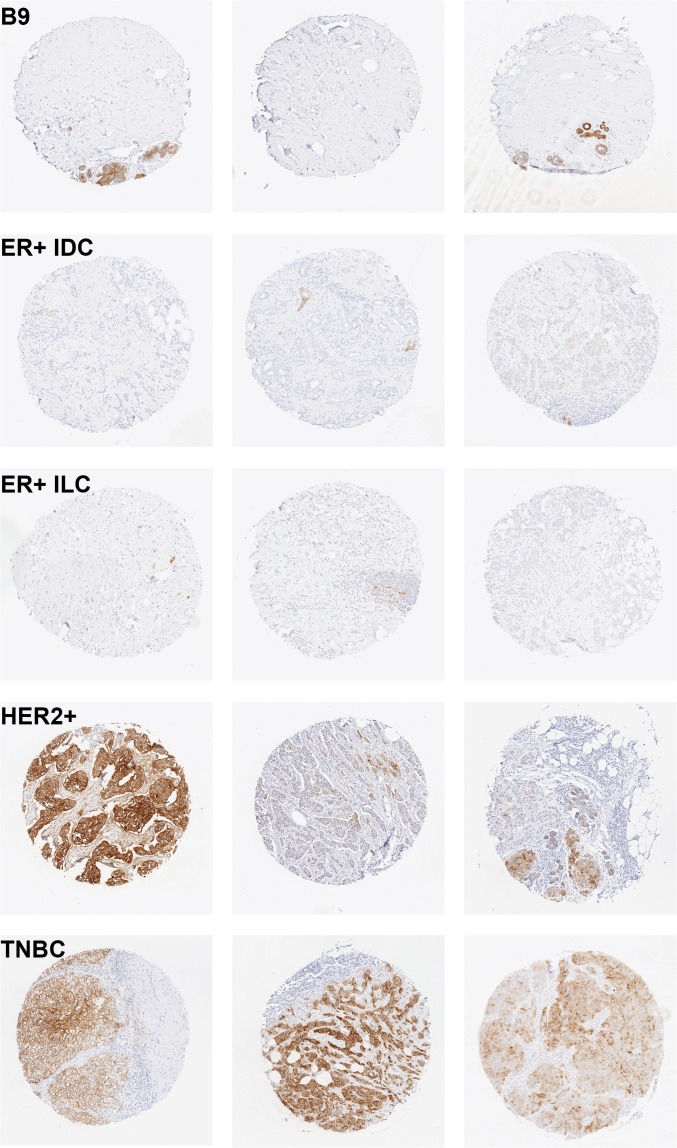
Representative FOLR1 staining from breast cancer subtypes. (A) benign breast tissue (B9) with negative staining (IHC score 0), (B) estrogen receptor positive invasive ductal carcinoma (ER+IDC) with negative staining (IHC score 0), (C) estrogen receptor positive invasive lobular carcinoma (ER+ILC) with negative staining (IHC score 0), (D) human epidermal growth factor receptor positive (HER2+) with strong 3+ staining (left core) and weak 1+ staining (middle/right cores), and (E) triple negative (TNBC) tumors with strong 3+ staining (left core) and moderate 2+ staining (middle/right cores).

**Fig 4:**
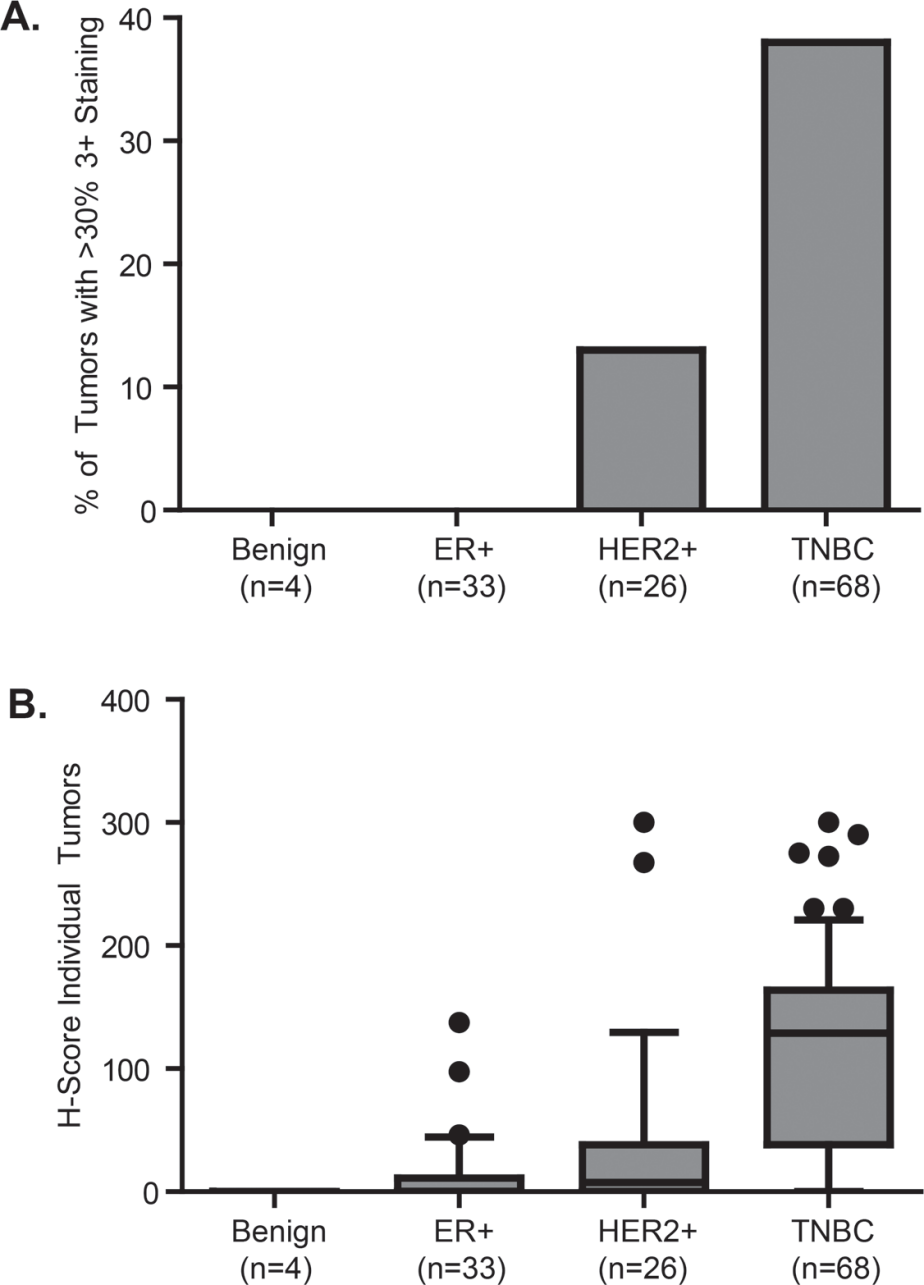
Analysis of FOLR1 immunohistochemical staining of breast cancer subtype tissue microarrays. TMAs comprised of 131 breast cancer tumors of different subtypes from patients with classified breast cancers subtypes that include DCIS (n = 4), ER+ (n = 33), HER2+ (n = 26) and TNBC (n = 68). TMAs were evaluated using high affinity FOLR1 antibody Mab 26B3.F2 and IHC staining intensities calculated as described in the “Materials and Methods”. (A). The percentage of breast tumors with > 30% 3+ staining by subtype. P-values calculated Mann-Whitney test of TNBC versus benign, ER+ and HER2+. (B). H-score distribution of FOLR1 expression. H-scores were calculated with the formula: *Hscore* = 3 * (% *at* 3 +) + 2 * (% *at* 2 +) + 1 * (% *at* 1 +) + 0 * (% *at* 0). *Statistical significance determined by Mann-Whitney test of TNBC versus benign, ER+ and HER2+ for both (A) and (B).

To confirm that FOLR1 protein expression correlated with the mRNA trends observed across breast cancer subtypes, we used immunohistochemistry to screen TMAs for membrane FOLR1. TMAs consisted of 131 tumors from patients with classified breast cancer subtypes that included DCIS (n = 4), ER+ (n = 33), HER2+ (n = 26) and TNBC (n = 68). TMAs were evaluated using mAb 26B3.F2, a high affinity antibody previously characterized to be specific for FOLR1 and suitable for FFPE immunohistochemistry [25]. A semi-quantitative scoring method was applied to assign staining intensities of negative, weak (1+), moderate (2+) and strong (3+). S5 Fig. illustrates the representative staining intensities across the breast cancer TMAs. The distribution of FOLR1 positivity varied substantially across subtypes of breast cancer. A significantly higher proportion of HER2+ and TNBC tumors were positive for FOLR1 as compared to ER+ tumors (Fig. 3). Using the criteria that > 30% of the tumor must exhibit 3+ membrane staining, the largest percentage of overexpressing tumors was represented by the triple negative subtype (Fig. 4A). This trend was also reflected with the H-score distribution among tumor subtypes (Fig. 4B). H-scores of triple negative tumors were significantly higher compared to the other breast cancer subtypes *(P*<0.001).

### FOLR1 expressing TNBC tumors are not associated with a particular TNBC subtype

**Fig 5:**
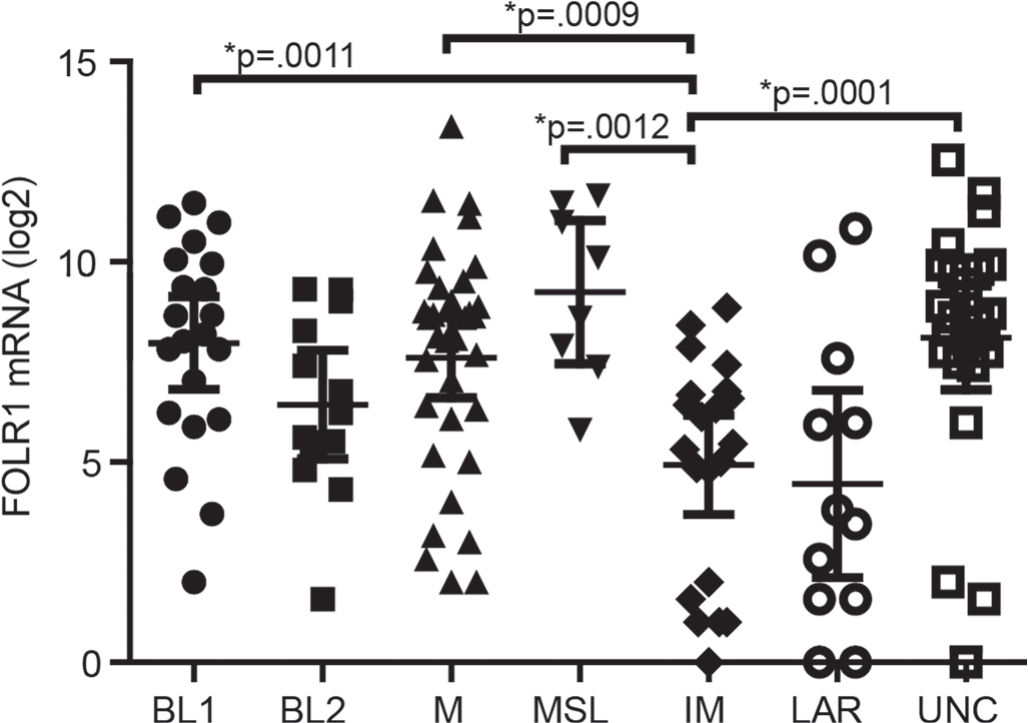
Distribution of FOLR1 tumor expression across TNBC tumor subtypes. *FOLR1* mRNA expression from 131 TN tumors from TCGA RNA-seq dataset were plotted versus the six TNBC subtypes that include basal-like (BL1 and BL2), immunomodulatory (IM), mesenchymal (M), mesenchymal stem-like (MSL), and luminal androgen receptor (LAR) reported by Lehman et al. [9]. Tumors were assigned to an unclassified group (UNC) if they had low correlations (<0.1) for any subtype or were similar between multiple subtypes (*P*<.05) [9]. Statistical significance across groups was determined by ANOVA Kruskal-Wallis test followed by Dunn’s Multiple Comparison Post test. *Significance at the *P* values shown was confirmed by Mann Whitney test of IM versus BL1, BL2, M, MSL, and LAR subtypes.

Our data indicates that FOLR1 expression is enriched in the TN population. We next determined if these tumors segregated into one of the six distinct TNBC subtypes according to *FOLR1* mRNA expression. These subtypes include basal-like (BL1, BL2), mesenchymal (M), mesenchymal stem-like (MSL), immunomodulatory (IM), and luminal androgen receptor (LAR) [9]. We analyzed *FOLR1* expression in 131 TN tumors from the TCGA RNA-seq dataset for which TN subtypes was previously predicted [35]. Fig. 5 shows that tumors with high *FOLR1* were distributed across the TN subtypes with no single TNBC subtype significantly enriched for higher FOLR1 expression. However, the IM subtype showed significantly lower median *FOLR1* expression as compared to BL1, M, MSL and unclassified subtypes. The lack of association of higher *FOLR1* with a TNBC subtype is also reflected by the presence of high *FOLR1* expressing tumors in the unclassified (UNC) group. Tumors assigned to this group had low correlations (<0.1) for any subtype or were similar between multiple subtypes (*P*<.05) [35].

### FOLR1 expression does not correlate with standard clinicopathologic features outside of hormonal status or HER2

To investigate whether FOLR1 expression correlated to common clinical features, we utilized the TCGA RNA-seq dataset due to its large sample size. Clinicopathologic characteristics of the tumors are shown in S2 Table. *FOLR1* expression was not associated with gender, tumor stage, or node status when compared across all breast cancer subtypes (S2 Table) or when the analysis was limited only to the TNBC subtype (S3 Table). We did observe a significant difference in median *FOLR1* expression between patients age (≤60, *P* = 0.002) when analysis was performed across all breast cancer subtypes (S2 Table), but this significance was lost when analysis was limited to TNBC tumors (S3 Table).

### Biological consequence of FOLR1 overexpression in TN tumors

**Fig 6:**
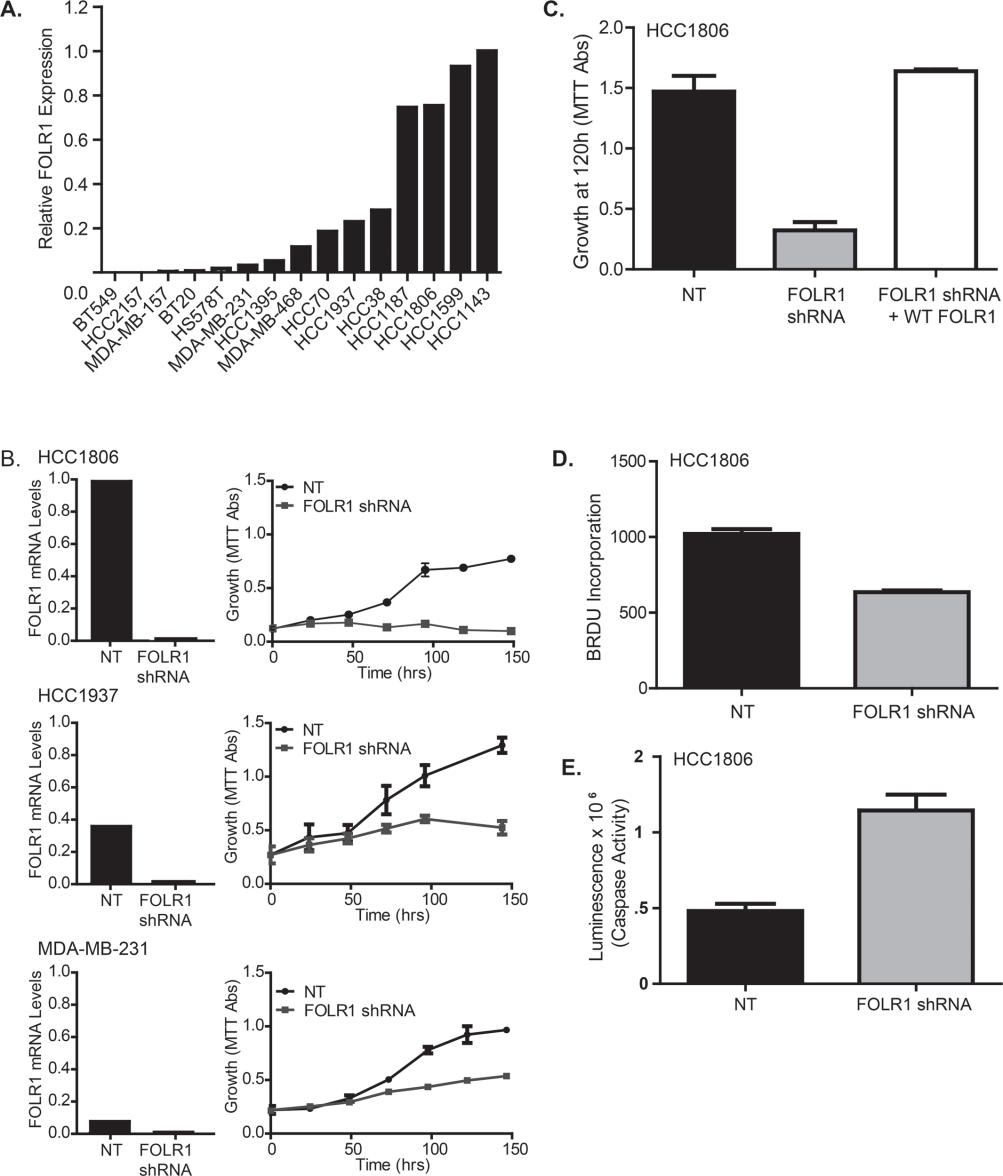
Expression of FOLR1 influences growth of TNBC breast cancer cell lines. (A). *FOLR1* mRNA abundance in 15 well-established TNBC cell lines as determined by qPCR. (B). FOLR1 expression was depleted by stable shRNA knockdown (KD) in a subset of established TNBC cell lines expressing high levels of *FOLR1* mRNA. The relative level of *FOLR1* KD was determined by quantitative PCR (left panel). The growth rates of FOLR1-depleted cells vs. control non-target cells were determined over 5 days using an MTT assay (right panel). Error bars represent ± SD. (C). Rescue of the growth phenotype in HCC1806 FOLR1 KD cells by forced overexpression of FOLR1. HCC1806 FOLR1 KD cells were infected with FOLR1 or NT retrovirus and selected for 7 days with 500ug/ml G418. Growth was measured by the MTT assay. Error bars represent ± SD. (D). BrdU incorporation in HCC1806 FOLR1 KD and NT cells after a 24 hr pulse. Data plotted as mean ± SD fluorescence represents relative BrdU incorporation. (E). Measurement of apoptosis in HCC1806 FOLR1 KD and NT cells using the luminescent Caspase-Glo 3/7 assay. Error bars represent ± SD.

Given that folate is essential for cell metabolism, we hypothesize that FOLR1 overexpression provides a growth advantage to the TN tumors. To evaluate this, we first asked whether inhibition of FOLR1 would disrupt cell growth of established TNBC cell lines. As with primary breast tumors, the abundance of *FOLR1* varies among TNBC cell lines, with a subset expressing high levels (Fig. 6A). Using RNA-interference, we depleted FOLR1 in three triple negative cell lines (MDA-MB-231, HCC1937, and HCC1806) expressing a range of *FOLR1* mRNA (Fig. 6B). TNBC cell lines with FOLR1 knockdown (KD) displayed reduced growth rates compared to their non-template control (Fig. 6B). The magnitude of the effect was proportional to their original mRNA expression level. This effect could be rescued by reconstitution with forced expression of wild type FOLR1 (Fig. 6C). We next evaluated whether the observed effects of FOLR1 on growth were due to changes on cell proliferation and/or apoptosis. Measurement of BrdU incorporation and apoptosis (caspase 3/7 activity) indicated the reduced growth rates in FOLR1 KD cells were due to both reduced DNA synthesis (Fig. 6D) and elevated apoptosis (Fig. 6E).

**Fig 7:**
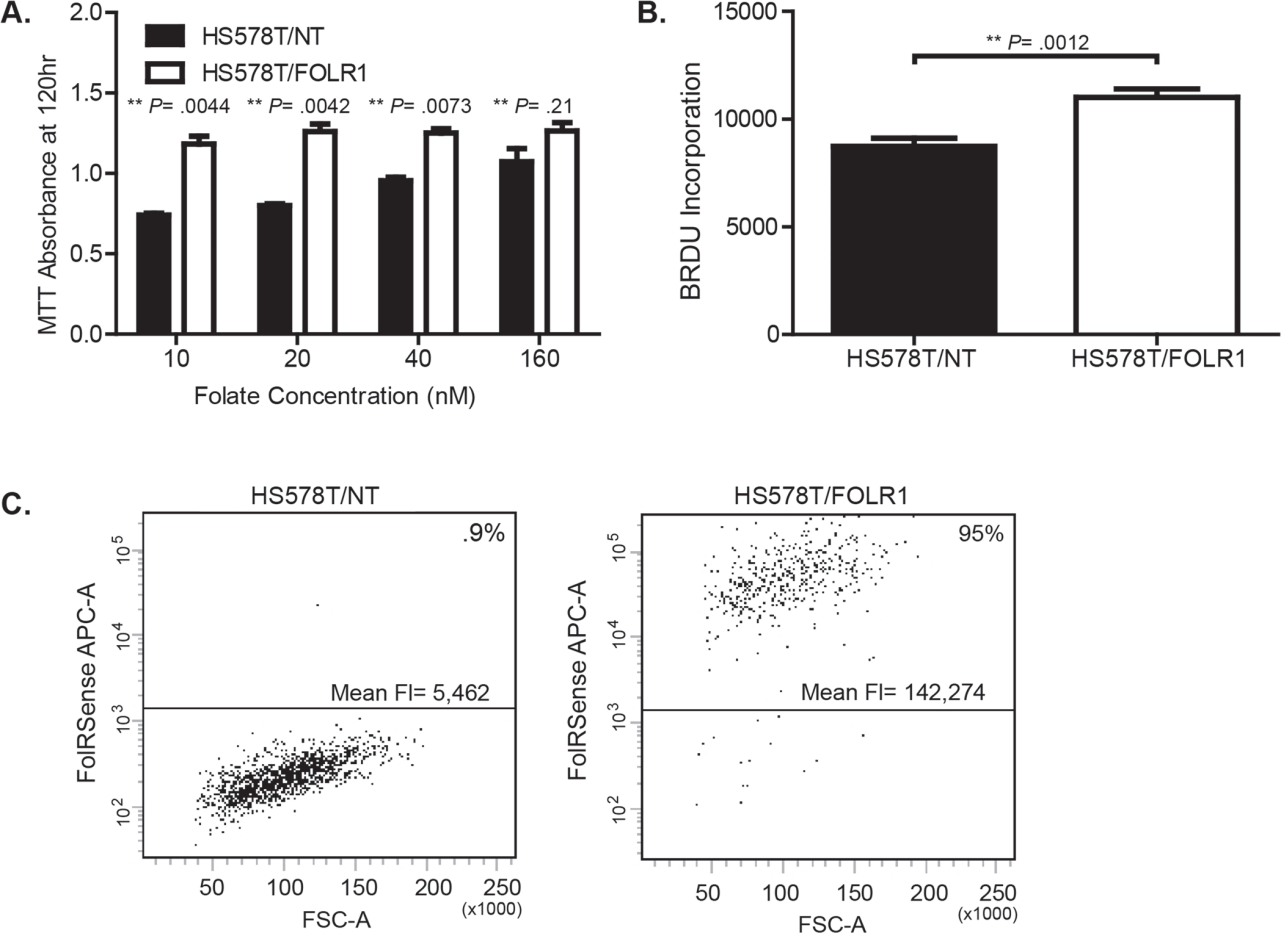
Folate receptor overexpression increases cell growth and folate uptake. (A). Growth of HS578T breast cancer cells engineered to stably overexpress FOLR1 or empty vector (NT). Cells were cultured in low (0–40 nM) and super-physiological (160 nM) concentrations of folic acid and growth determined after 120 hr using the MTT assay. Error bars represent ± SD. **Statistical significance calculated by two sided unpaired t-test, assuming unequal variances. (B). BrdU incorporation in HS578T/NT and HS578T/FOLR1 cells grown in 10 nM folic acid. **Statistical significance calculated by two sided unpaired t-test, assuming unequal variances. (C). Folate uptake in HS578T/NT versus HS578T/FOLR1 cells. Briefly, flow cytometry was used to measure internalized fluorescent folate (fluorescent tagged folate agent, FolateRSense 690) after 1 hr of exposure. Dot plots show forward scatter area (FSC-A) vs. FolateRSense fluorescence (APC). The percent of cells with internalized fluorescent folate and the mean fluorescence per cell type are shown.

To confirm the requirement of FOLR1 for cellular growth by a different means, we overexpressed the folate receptor in HS578T cells, a TNBC cell line with lower levels of *FOLR1* (Fig. 6A). FOLR1 overexpression promoted growth in sub- physiological concentrations (10–40 nM) of extracellular folate (Fig. 7A) compared to control. The increased growth rate of FOLR1 overexpressing cells at sub- physiological concentrations of folate was reflected in an increase in DNA synthesis (Fig. 6B). No significant difference in growth was observed among cells grown in supra-physiological concentrations of folate (160 nM). To determine whether the growth advantage induced by FOLR1 overexpression was due to an increase in folate uptake, we measured folate internalization by flow cytometry using a fluorescent tagged folate agent, FolateRSense 690 (Perkin Elmer). After 1 hr of exposure to FolateRSense in folate- free media, cells were washed and acid stripped to remove surface bound folate, and internalized fluorescent folate was measured via flow cytometry. HS578T cells overexpressing FOLR1 displayed significantly greater folate uptake as shown by an increase in the number of cells with internalized FolateRSense (0.9% vs, 95%) and an increase in mean fluorescence (Fig. 7C).

## Discussion

Our central objective was to examine FOLR1 expression patterns in clinically relevant molecular subtypes of breast cancer (ER+, HER2+, and TNBC) to identify whether there was differential expression, and to explore a potential mechanistic role of FOLR1 in breast cancer cells. Prior to our work, studies investigating FOLR1 expression in breast cancer had been largely limited to semi-quantitative immunohistochemistry [23–25]. Caveats to this approach include limited sample size and inter-laboratory variability of immunohistochemistry staining arising from technical issues (antibodies, protocols, scoring etc.). Our first objective was therefore to use mRNA-seq data to compose a detailed expression pattern of *FOLR1* mRNA among breast cancer subtypes. This enabled us to screen multiple patient cohorts to achieve a larger sample number. It should be noted that the normalization of mRNA seq-data across tumor datasets is an issue, since we are comparing samples prepared with different library and sequencing platforms, and there are no well-established criteria for compensating for such issues. As comparing the counts among data sets is not suitable, we compared overall trends. We observed that subpopulations of triple negative tumors expressed high levels of *FOLR1* mRNA, and overall had the highest median abundance of *FOLR1* of any subtype. Conversely, ER+ tumors expressed significantly lower levels of *FOLR1* mRNA than ER-. These associations were supported by our immunohistochemical analyses, conducted by experienced pathologists, with the largest H-scores and percent 3+ positive tumors of any subtype represented by triple negatives, and lowest by ER+ tumors. In general, the protein expression data for FOLR1 reflected that observed for the mRNA across the breast cancer subtypes. One exception is the observation that small subpopulations of Luminal A and Luminal B tumors expressed high levels of *FOLR1* mRNA (Fig. 1A and 1B), although high level FOLR1 protein staining was not observed in our TMA samples (> 30% 3+ staining, Fig. 4A). Discordance between mRNA and protein may reflect the higher sample size of the TCGA mRNA-seq dataset. Alternatively, this disconnect may represent possible posttranslational control of FOLR1 mRNA or in expression of a soluble (non-membrane bound) form of FOLR1. Some discordance between mRNA and protein may also reflect heterogeneous expression of FOLR1 within the tumor. TMAs are a small snapshot of the tumor and therefore susceptible to potential intra-tumor heterogeneity. Such heterogeneity would be not observed in mRNA levels as RNA was isolated from a larger section of the tumor.

Overall, our data indicate FOLR1 expression is higher in the TNBC subtype in general, which includes a subpopulation of particularly “high” FOLR1 expressing tumors. Given that triple negative breast cancer is a heterogeneous disease, it is tempting to speculate that tumors expressing high levels of FOLR1 represent a specific subtype. Lehman et al. [9] recently classified triple negative cancers into six subtypes (BL1, BL2, M, MSL, IM and LAR) based on gene expression and ontology signatures. However, we found that tumors with high *FOLR1* expression did not segregate into any particular subtype within triple negative tumors, suggesting tumors expressing high FOLR1 are quite heterogeneous in nature. Interestingly, the immunomodulatory subtype tended to show significantly lower FOLR1 levels as compared to several other TN subtypes (BL1, M, MSL, and UNC). The significance of the association between low FOLR1 levels and the immunomodulatory subtype is unclear. Further work is needed to confirm and extend any possible link of FOLR1 with immune responses.

Our laboratory found no association of FOLR1 expression in triple negative tumors with common or standard clinicopathological parameters such as age, tumor stage and nodal status. These findings are consistent with of those of O’Shannessy et al. [25] but contrast with those of Zhang and coworkers [24], who reported a significant association of FOLR1 with higher nodal stage (*P* = .03, N0/N1 versus N2/N3). We believe the discrepancy arises from the use of different expression criteria for correlating FOLR1. Zhang et al. [24] compared nodal stage to FOLR1 protein positivity, a measure of whether the tumor is positive for FOLR1 (>10% membrane staining at any intensity). This is in contrast to our quantitative approach that examines mRNA expression values of *FOLR1*.

Our observation of a negative correlation of FOLR1 with estrogen receptor expression is consistent with previous findings of Rochman et al. [36] and O’Shannessy et al. [25] who reported significantly higher expression of FOLR1 in ER- tumors than ER+ tumors. One possible explanation for this relationship is that overall abundance of FOLR1 may, at least in part, be controlled by the levels of the estrogen receptor, as well as estrogen and estrogen receptor antagonists active within ER+ tumors. A study by Kelley et al. [37] demonstrated that FOLR1 expression can be repressed in the presence of estradiol, and de-repressed in the presence of anti-estrogens such as tamoxifen. This repression stems from regulation of the estrogen responsive P4 promoter of FOLR1. An alternative promoter, termed P1, was found to be estrogen-independent [36]. Interestingly, our laboratory has found that HER2+ and ER+ cells express more of FOLR1 isoform 4, the isoform driven by the estrogen responsive P4 promoter (S4 Table). As these cell types express ER and have lower FOLR1 levels, it is likely that FOLR1 expression is controlled by estrogen dependent regulation of the P4 promoter. Likewise, we found that TNBC cells express isoforms driven by both the estrogen independent P1 (Isoform 7) and dependent P4 (isoform 4) promoters of FOLR1 (S4 Table). As such, these studies suggest the regulation of FOLR1 expression may be complex, depending on the particular breast cancer subtype, and involve both alternative promoter utilization and estrogen–dependent regulation.

The finding that a significant number of triple negative cancers express abundant FOLR1 raises the question of the biological consequence of FOLR1 overexpression. To this end, we show that altering FOLR1 expression by shRNA knockdown can influence the growth of triple negative breast cancer cells. Over-expression of FOLR provides a growth advantage to cells, particularly in a low folate environment, where FOLR1 increases folate uptake into the cell. It is logical to hypothesize that overexpression in breast cancer cells may be a mechanism to provide adequate intracellular folate concentrations in a folate deprived microenvironment and/or to meet increased metabolic needs of the tumor cell.

TN breast cancer has thus far been a diagnosis of exclusion and treatment options for these patients are limited due to an absence of potential targeted therapy. The finding that a significant number of TNBCs express abundant FOLR1 suggests an important population of patients may benefit by targeting FOLR1 positive cells. It should also be emphasized that high level FOLR1 expression is not limited to TNBC tumors. A small subset of ER+ tumors (mostly Luminal B) expresses abundant *FOLR1*, and a somewhat larger subset of HER2+ tumors exhibit a similar molecular phenotype. These populations may also benefit from FOLR1 targeted therapy. Anti-Folate receptor targeted therapies such as farletuzumab (monoclonal antibody), vintafolide (folate- small molecule conjugated to the chemotherapeutic drug vinblastine) and dendritic cell based vaccines are in various phases of clinical trials for treatment of ovarian and lung cancer, and are logical choices for investigation in triple negative tumors.

## Conclusions

Our study reports that FOLR1 expression, as assessed by RNA-sequencing and immunohistochemistry, varies widely among subtypes of breast cancer. Importantly, high level expression of FOLR1 is significantly associated with triple negative/basal tumors compared to ER+ and HER2+ tumors. Expression of FOLR1 influences the growth of triple negative cells, and its overexpression promotes folate uptake and provides a selective growth advantage in low folate conditions. Therefore, patients with triple negative tumors expressing high FOLR1 represent an important subpopulation that may benefit from targeted anti-FOLR1 therapy.

## Supporting Information

S1 Fig Violin plot of FOLR1 expression across clinically relevant breast cancer subtypes. Log2 transformed data of FOLR1 abundance from a TCGA dataset of 691 breast cancers classified as basal, Luminal A, Luminal B, and HER2+. Bars represent 95% confidence levels for difference between the means.
(TIF)

S2 Fig Expression of FOLR1 in basal versus triple negative tumors. FOLR1 mRNA expression values (log2) of TCGA RNA-seq dataset (Fig. 1) representing the classification of the basal population. Expression values (log2) of all basal tumors (TNBC + non-TNBC) was plotted against the TNBC only subpopulation (85% of all basal tumors) and the non-TNBC subpopulation (15% of all basal tumors).
(TIF)

S3 Fig RNA-seq analysis of FOLR1 expression in ER- basal tumors versus ER+ luminal breast cancers. Data is plotted as FOLR1 mRNA abundance (log2) from TCGA breast cancers classified as being ER- Basal versus ER+ Luminal A and ER+ Luminal B. Bars represent 95% confidence levels for difference between the means. Statistical significance was calculated by two sided unpaired t-test, assuming unequal variances. Significance was *P* <0.0001 for basal versus Luminal A and Luminal B.
(TIF)

S4 Fig Microarray analysis of FOLR1 expression in ER+ versus ER- tumors. We analyzed a cohort of 129 primary breast cancer gene expression profiles generated using the Affymetrix U133plus2 platform and downloaded from Gene Expression Omnibus (GEO accession number: GSE5460) [33]. Log2 transformed data is shown with bars indicating 95% confidence levels for differences between the means. *Statistical significance calculated by two sided unpaired t-test, assuming unequal variances.
(TIF)

S5 Fig FOLR1 antibody membrane staining intensities in a breast cancer tissue. Immunohistochemistry (IHC) was performed using FFPE tissue and FOLR1 antibody mAB 26B3.F2 as described in the “Materials and Methods”. Membrane staining as scored as negative (IHC score 0), weak (IHC score 1+), moderate (IHC score 2+), and strong (IHC score 3+).
(TIF)

S1 Table Normalized RNA Seq. Values of FOLR1 from Triple Negative Tumors: Mayo Clinic-Jacksonville. (DOCX)

S2 Table Correlation of FOLR1 expression and clinicopathologic features in all breast cancer subtypes. (DOCX)

S3 Table Correlation of FOLR1 expression and clinicopathologic features in TNBC. (DOCX)

S4 Table qPCR ΔCT values of FOLR1 isoforms from breast cancer cell lines. FOLR1 mRNA expression values (ΔCT) are presented normalized to the endogenous control POLR2A (ΔCT = CT of FOLR1-CT of POLR2A). Lower values represent higher abundance.
(DOCX)
