# Correction: Folate Receptor-α (FOLR1) Expression and Function in Triple Negative Tumors

**DOI:** 10.1371/journal.pone.0127133

**Published:** 2015-04-30

**Authors:** Brian M. Necela, Jennifer A. Crozier, Cathy A. Andorfer, Laura Lewis-Tuffin, Jennifer M. Kachergus, Xochiquetzal J. Geiger, Krishna R. Kalari, Daniel J. Serie, Zhifu Sun, Alvaro Moreno-Aspitia, Daniel J. O’Shannessy, Julia D. Maltzman, Ann E. McCullough, Barbara A. Pockaj, Heather E. Cunliffe, Karla V. Ballman, E. Aubrey Thompson, Edith A. Perez

The tenth author’s name is spelled incorrectly. The correct name is: Alvaro Moreno-Aspitia.

The Academic Editor field is missing from the published paper. The name of the Academic Editor is: Aamir Ahmad, Wayne State University School of Medicine, United States.
