# Supplementary figures and images for: Folate Receptor-α (FOLR1) Expression and Function in Triple Negative Tumors
Source: PLoS One. 2015 Mar 27;10(3):e0122209. doi: 10.1371/journal.pone.0122209 (PMC4376802; doi:10.1371/journal.pone.0122209)

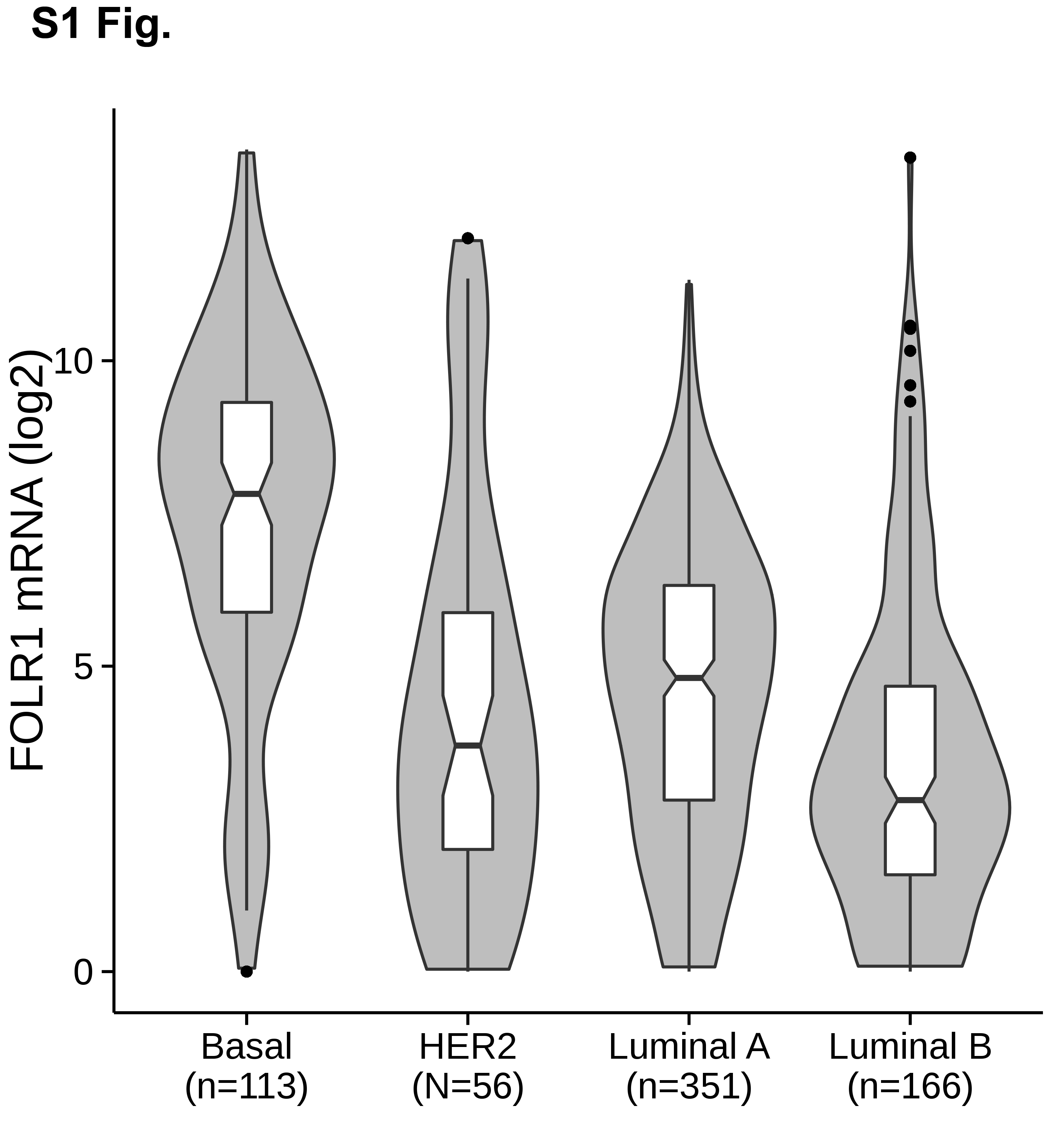

Supplement: S1 Fig — Log2 transformed data of FOLR1 abundance from a TCGA dataset of 691 breast cancers classified as basal, Luminal A, Luminal B, and HER2+. Bars represent 95% confidence levels for difference between the means. (TIF) [file pone.0122209.s001.tif]

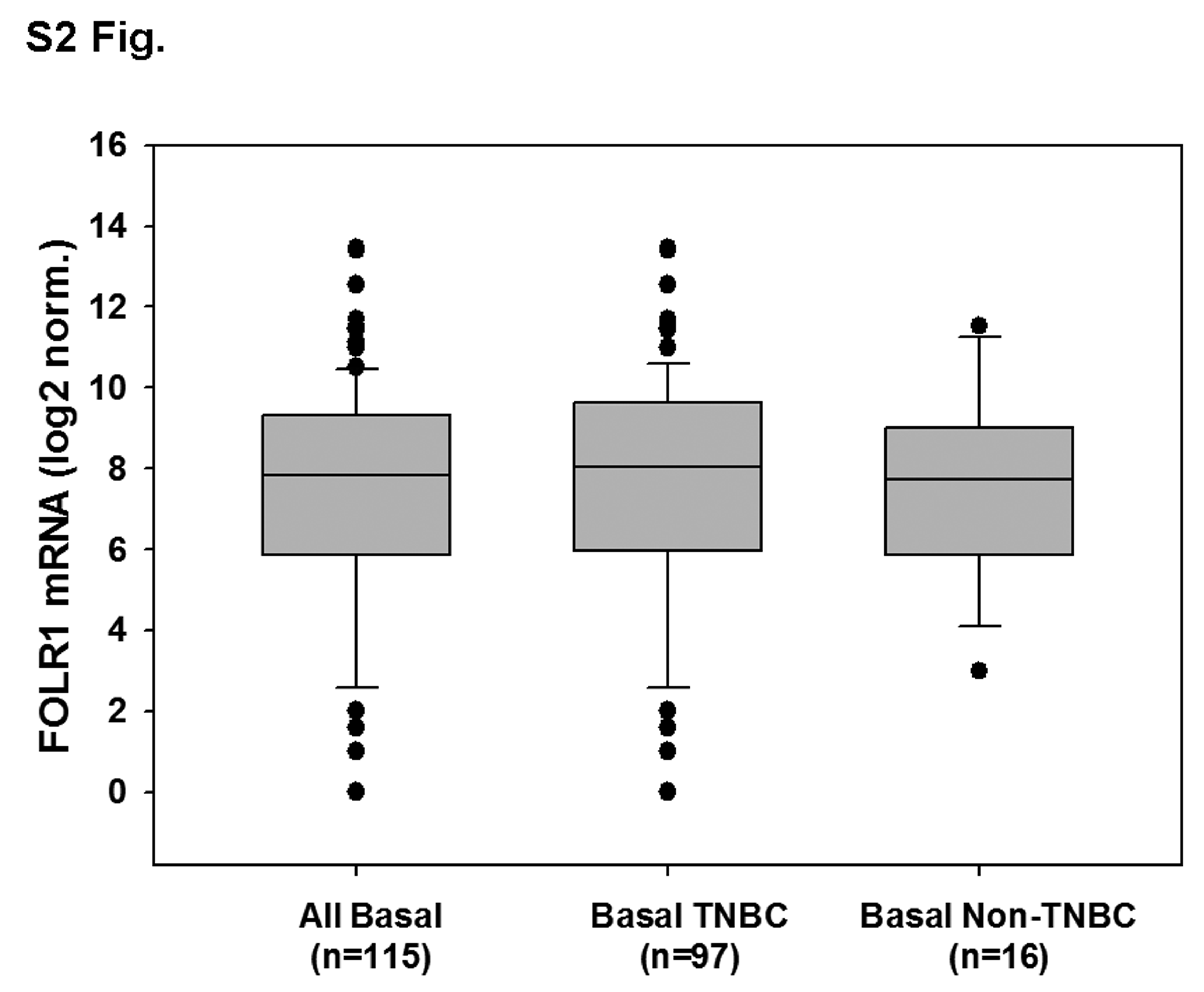

Supplement: S2 Fig — FOLR1 mRNA expression values (log2) of TCGA RNA-seq dataset (Fig. 1) representing the classification of the basal population. Expression values (log2) of all basal tumors (TNBC + non-TNBC) was plotted against the TNBC only subpopulation (85% of all basal tumors) and the non-TNBC subpopulation (15% of all basal tumors). (TIF) [file pone.0122209.s002.tif]

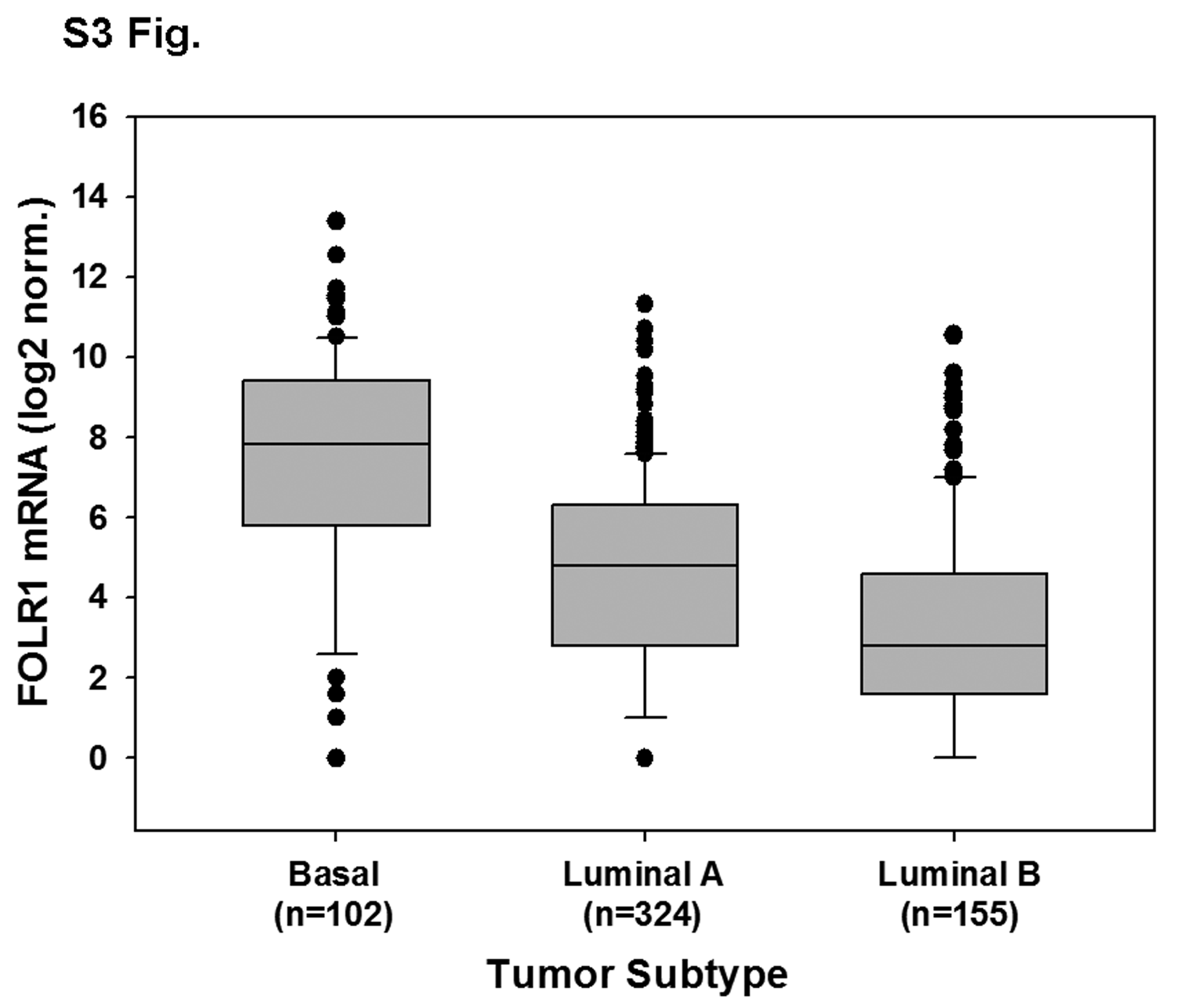

Supplement: S3 Fig — Data is plotted as FOLR1 mRNA abundance (log2) from TCGA breast cancers classified as being ER- Basal versus ER+ Luminal A and ER+ Luminal B. Bars represent 95% confidence levels for difference between the means. Statistical significance was calculated by two sided unpaired t-test, assuming unequal variances. Significance was P <0.0001 for basal versus Luminal A and Luminal B. (TIF) [file pone.0122209.s003.tif]

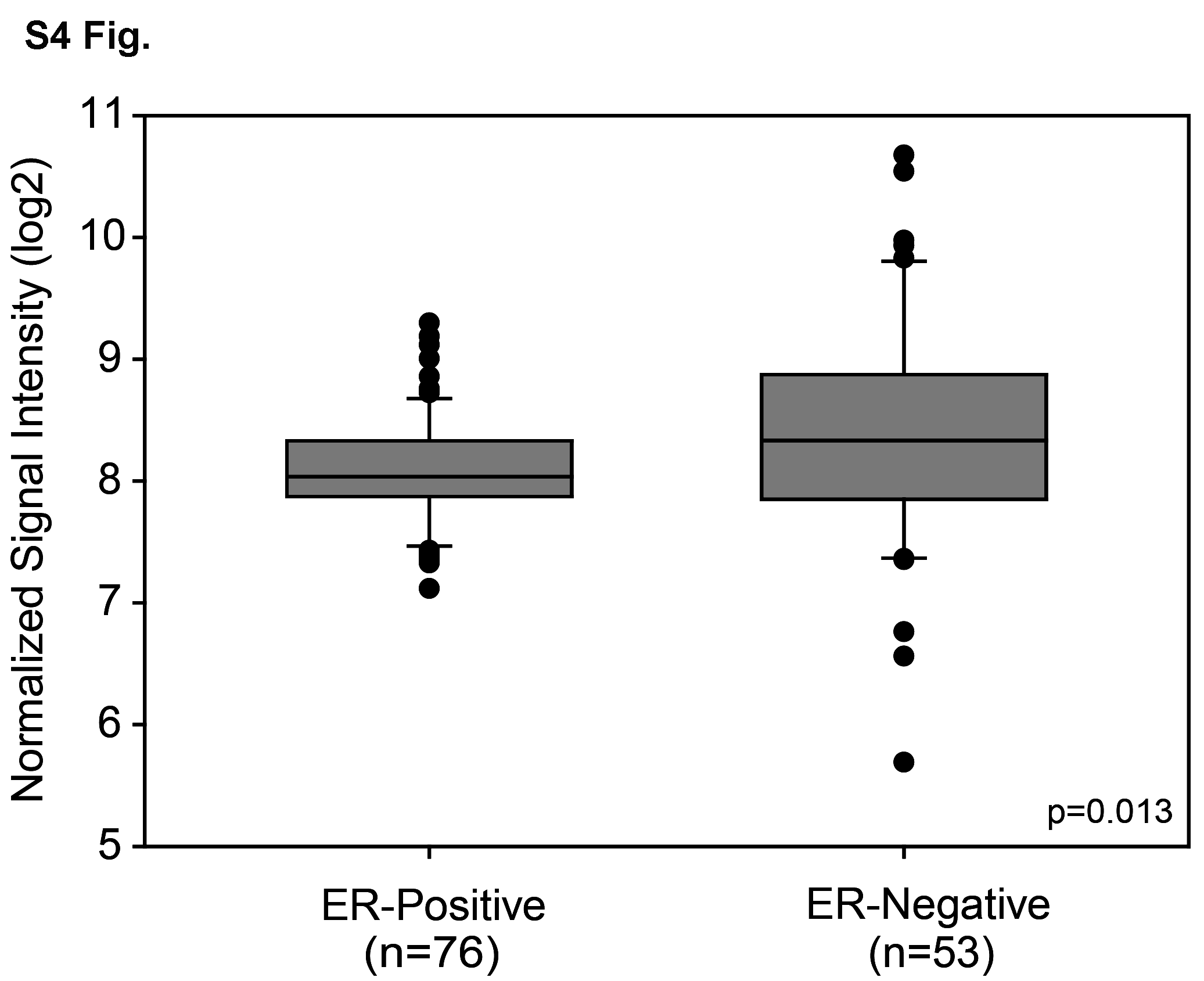

Supplement: S4 Fig — We analyzed a cohort of 129 primary breast cancer gene expression profiles generated using the Affymetrix U133plus2 platform and downloaded from Gene Expression Omnibus (GEO accession number: GSE5460) [33]. Log2 transformed data is shown with bars indicating 95% confidence levels for differences between the means. *Statistical significance calculated by two sided unpaired t-test, assuming unequal variances. (TIF) [file pone.0122209.s004.tif]

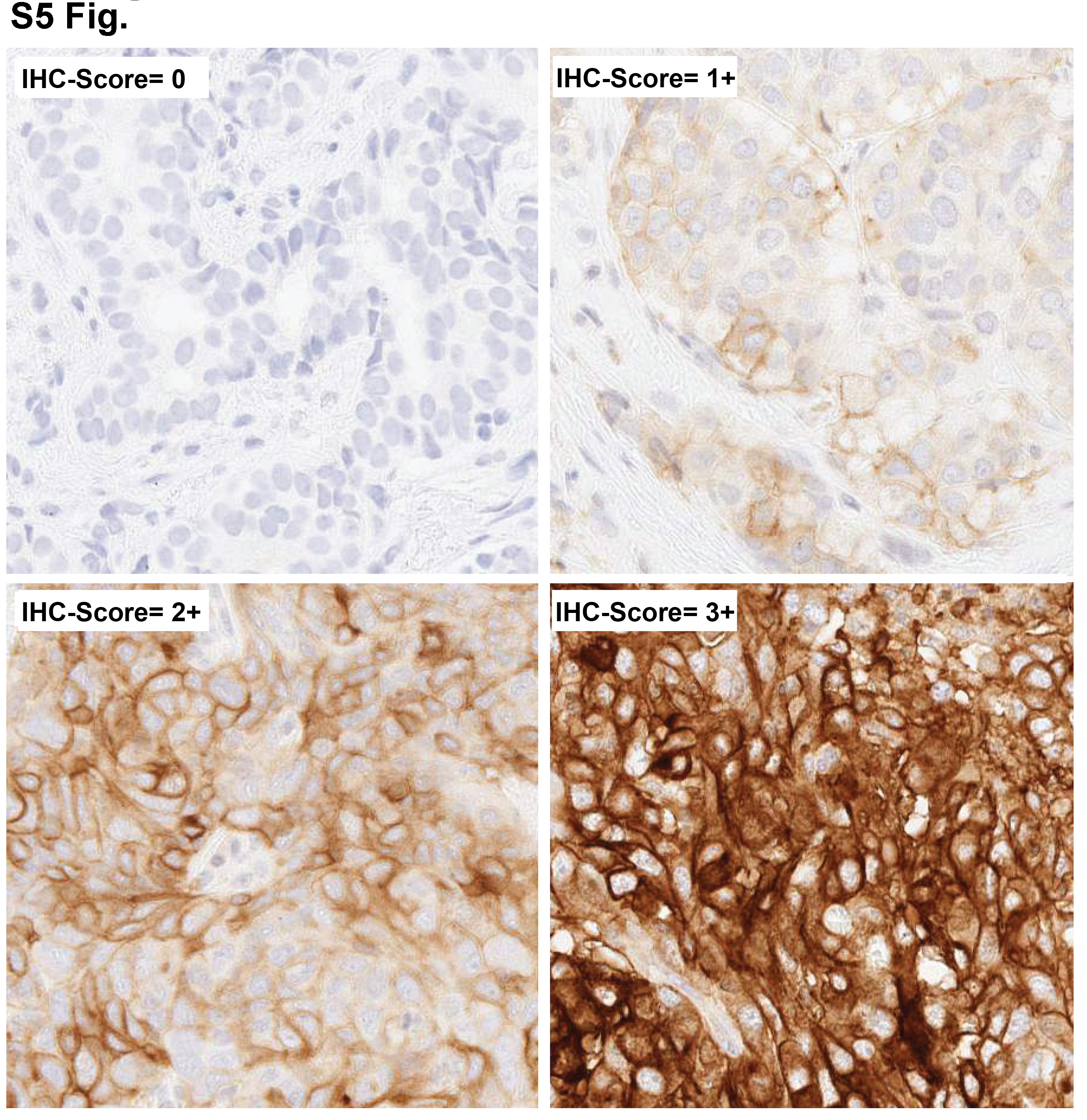

Supplement: S5 Fig — Immunohistochemistry (IHC) was performed using FFPE tissue and FOLR1 antibody mAB 26B3.F2 as described in the “Materials and Methods”. Membrane staining as scored as negative (IHC score 0), weak (IHC score 1+), moderate (IHC score 2+), and strong (IHC score 3+). (TIF) [file pone.0122209.s005.tif]
