# Supplementary material for: Folate Receptor-α (FOLR1) Expression and Function in Triple Negative Tumors
Source: PLoS One. 2015 Mar 27;10(3):e0122209. doi: 10.1371/journal.pone.0122209 (PMC4376802; doi:10.1371/journal.pone.0122209)
Supplement: S1 Table — (DOCX) [file pone.0122209.s006.docx]

**S1 Table. Normalized RNA Seq. Values of FOLR1 from Triple Negative Tumors: Mayo Clinic-Jacksonville**

| **Sample ID** | **FOLR1 (log2)** |  | **Sample ID** | **FOLR1 (log2)** |  | **Sample ID** | **FOLR1 (log2)** |
| --- | --- | --- | --- | --- | --- | --- | --- |
| B1194A_L149 | 0 |  | B1927A_L156 | 7.5392 |  | B478_L113 | 5.7549 |
| B1232A_L148 | 6.0661 |  | B1967A_L155 | 1 |  | B551_L141 | 3.3219 |
| B1269B_L147 | 1 |  | B1995B_L154 | 3 |  | B577A_L140 | 1 |
| B1287A_L146 | 0 |  | B2218A_L153 | 2 |  | B600C_L139 | 0 |
| B1451B_L145 | 0 |  | B2482_L152 | 2 |  | B775A_L138 | 4.7549 |
| B1620A_L144 | 7.3487 |  | B2763A_L151 | 2.8074 |  | B826A_L150 | 8.8611 |
| B1662A_L143 | 7.3309 |  | B339A_L118 | 4.7004 |  | B880A_L137 | 7.0444 |
| B1704A_L142 | 7.1085 |  | B365A_L117 | 7.7074 |  | B920_L120 | 4.5236 |
| B1851A_L159 | 7.6294 |  | B380_L116 | 7.3837 |  | B941B_L119 | 4.858 |
| B1860A_L158 | 0 |  | B449_L115 | 5.1293 |  |  |  |
| B1900C_L157 | 6.2095 |  | B456_L114 | 6.6582 |  |  |  |
