# Supplementary material for: Folate Receptor-α (FOLR1) Expression and Function in Triple Negative Tumors
Source: PLoS One. 2015 Mar 27;10(3):e0122209. doi: 10.1371/journal.pone.0122209 (PMC4376802; doi:10.1371/journal.pone.0122209)
Supplement: S2 Table — (DOCX) [file pone.0122209.s007.docx]

**S2 Table. Correlation of FOLR1 expression and clinicopathologic features in**

**all breast cancer subtypes**

| **Variable No. Patients FOLR1 mRNA Mann-Whitney Test**  **(Mean ± SD) *(P* value)** | | | |
| --- | --- | --- | --- |
| Age (years) |  |  |  |
| <60 | 410 | 5.25±2.93 | .0020 |
| ≥60 | 330 | 4.57±2.97 |  |
|  |  |  |  |
| Gender |  |  |  |
| Male | 7 | 4.96±2.96 | .0673 |
| Female | 733 | 2.89±2.44 |  |
|  |  |  |  |
| Tumor Stage |  |  |  |
| T1 | 187 | 5.07±2.87 | >.05 |
| T2 | 406 | 4.81±3.03 |  |
| T3 | 73 | 4.86±2.70 |  |
| T4 | 26 | 4.00±2.60 |  |
|  |  |  |  |
| Lymph Node Status |  |  |  |
| N0 | 322 | 4.82±2.94 | >.05 |
| N1 | 233 | 4.83±2.99 |  |
| N2 | 90 | 4.95±2.91 |  |
| N3 | 37 | 5.06±3.04 |  |
