# Supplementary material for: Folate Receptor-α (FOLR1) Expression and Function in Triple Negative Tumors
Source: PLoS One. 2015 Mar 27;10(3):e0122209. doi: 10.1371/journal.pone.0122209 (PMC4376802; doi:10.1371/journal.pone.0122209)
Supplement: S3 Table — (DOCX) [file pone.0122209.s008.docx]

**S3 Table. Correlation of FOLR1 expression and clinicopathologic features in**

**TNBC**

| **Variable No. Patients FOLR1 mRNA Mann-Whitney Test**  **(Mean ± SD) *(P* value)** | | | |
| --- | --- | --- | --- |
| Age (years) |  |  |  |
| <60 | 67 | 7.52±2.86 | .642 |
| ≥60 | 30 | 7.69±2.89 |  |
|  |  |  |  |
| Gender |  |  |  |
| Male | 0 |  |  |
| Female | 97 | 7.57±2.86 |  |
|  |  |  |  |
| Tumor Stage |  |  |  |
| T1 | 23 | 8.34±2.88 | >.05 |
| T2 | 59 | 7.38±2.92 |  |
| T3 | 9 | 7.04±2.39 |  |
| T4 | 2 | 6.02±.298 |  |
|  |  |  |  |
| Lymph Node Status |  |  |  |
| N0 | 64 | 7.33±2.85 | >.05 |
| N1 | 22 | 7.86±266 |  |
| N2 | 6 | 9.03±2.52 |  |
| N3 | 4 | 7.02±3.55 |  |
