# Supplementary material for: Folate Receptor-α (FOLR1) Expression and Function in Triple Negative Tumors
Source: PLoS One. 2015 Mar 27;10(3):e0122209. doi: 10.1371/journal.pone.0122209 (PMC4376802; doi:10.1371/journal.pone.0122209)
Supplement: S4 Table — FOLR1 mRNA expression values (ΔCT) are presented normalized to the endogenous control POLR2A (ΔCT = CT of FOLR1-CT of POLR2A). Lower values represent higher abundance. (DOCX) [file pone.0122209.s009.docx]

**S4 Table: qPCR ΔCT values of FOLR1 isoforms from breast cancer cell lines**

| **Cell Line** | **Subtype** | **Total FOLR1** | **FOLR1 Isoform 4** | **FOLR1 Isoform 7** |
| --- | --- | --- | --- | --- |
| HCC1806 | TN | -.097 | 4.44 | 6.47 |
| MDA-MB-231 | TN | 3.03 | 5.46 | 4.16 |
| HCC1954 | HER2+ | 0.55 | 1.78 | 7.77 |
| MCF-7 | ER+ | 1.95 | 3.75 | 12.33 |

FOLR1 mRNA expression values (ΔCT) are presented normalized to the endogenous control

POLR2A (ΔCT=CT of FOLR1-CT of POLR2A). Lower values represent higher abundance.
